# Investigation Into the Effect of COVID-19 Infection on Length of Hospital Stay and Mortality in Patients With Rheumatoid Arthritis

**DOI:** 10.7759/cureus.22685

**Published:** 2022-02-28

**Authors:** Kevin Thompson, Ami Shah, Adam Grunbaum, Olu Oyesanmi

**Affiliations:** 1 Internal Medicine, Citrus Memorial Hospital, Inverness, USA; 2 Internal Medicine, University of South Florida, Regional Medical Center Bayonet Point, Hudson, USA; 3 Rheumatology, University of South Florida, Regional Medical Center Bayonet Point, Hudson, USA; 4 Internal Medicine, Oak Hill Hospital, Brooksville, USA

**Keywords:** immune-mediated inflammatory disorder, rheumatology, covid, covid-19, rheumatoid arthritis

## Abstract

Background

SARS-CoV-2 (COVID-19) is a positive-stranded ribonucleic acid (RNA) virus of the coronavirus family, which has resulted in one of the most serious pandemics, with more than 14 million cases confirmed globally. Rheumatoid arthritis (RA) is estimated to be prevalent in 0.5-1% of the U.S. population. So far, there has been little evidence of COVID-19 infection and its propensity to result in increased mortality or length of hospital stay in patients with RA. To contribute to this body of literature, this study will assess the degree to which COVID-19 is associated with increased mortality and length of hospital stay in patients with RA while also taking into account these patients' comorbidities.

Methods

Our retrospective study included 14,180 patients (age >18, median 58, range 18-90) who tested positive for COVID-19 or were assumed to have COVID-19 infection from January 1^st^, 2020, through July 31^st^, 2020. Patients were grouped based on the diagnosis of RA and COVID-19 infection versus those without RA. Patients who were diagnosed with systemic lupus erythematosus (SLE), chronic obstructive pulmonary disease, and hypertension were excluded. Covariates included age, body mass index (BMI), race, sex, maximum C-reactive protein value, maximum D-dimer value, and comorbid diabetes mellitus. Outcome measures were length of hospital stay (LOS), in-hospital mortality, intensive care unit (ICU) admission, ICU LOS, mechanical ventilation, time on mechanical ventilation, and discharge to hospice. The logistic regression model was used to estimate the probability of in-hospital mortality, ICU admission, placement on mechanical ventilation, discharge to hospice, and in-hospital mortality related to home anti-inflammatory use when comparing patients with RA and COVID-19 infection to COVID-19 infected patients without RA.

Results

Of the total 14,180 patients (males 57.1%, females 42.9%), 159 patients (1.1%), had a diagnosis of RA. There was no significant association between RA and hospital LOS, ICU admission, ICU LOS, LOS on mechanical ventilation, or discharge to hospice among those infected with COVID-19. Yet, RA was associated with higher mortality (OR: 1.65; 95% CI: 1.07-2.53; p=0.02) and placement on mechanical ventilation (OR: 1.82; 95% CI: 1.22-2.71; p<0.01) amidst patients infected with COVID-19.

Conclusion

This study suggests that patients with RA and COVID-19 have a significantly increased likelihood of in-hospital mortality and placement on mechanical ventilation. While challenging to realize in a pandemic situation, large studies nationwide are necessary to improve our understanding of COVID-19 infection in patients diagnosed with RA.

## Introduction

SARS-CoV-2 is a positive-stranded ribonucleic acid (RNA) virus of the coronavirus family, which has resulted in one of the most serious pandemics, with more than 14 million cases confirmed globally. The range of associated symptoms is diverse; the most frequently observed are cough, fever, myalgia, headache, and dyspnea [1]. Some of the most common medical comorbidities associated with severe illness and mortality are cardiovascular disease, diabetes mellitus, hypertension, chronic lung disease, cancer, and chronic kidney disease [2-5]. Rheumatoid arthritis (RA) is estimated to be prevalent in 0.5-1% of the U.S. population [6]. As seen in the population-based study by Doran [7], there is an increased risk of serious infection from diseases in patients with RA compared to normal subjects (rate ratio [RR]: 1.53, 95% CI: 1.41-1.65). The increased risk of infection was largely attributed to the fact that patients with RA have an immune system that does not function optimally. This increased rate of infection is dependent on the degree of RA disease activity, which is evident in the study by Au and colleagues demonstrating that for each 0.6 unit rise in Disease Activity Score 28, there was a parallel increase in infection requiring hospitalization of 25% (incident rate ratio 1.25, p=0.03) [8].

In addition to the nature of RA's effect on immune function in itself, one must also account for the influence of standard RA treatment such as systemic corticosteroids, biologic disease-modifying antirheumatic drugs (bDMARDs), and conventional synthetic disease-modifying antirheumatic drugs (csDMARDs). The predilection of systemic corticosteroids to increase the risk of viral infection in patients with RA is appreciated in a retrospective-cohort study by Widdifield and associates, which revealed an increased risk of herpes-zoster infection with an incidence rate of 8.54 cases per 1,000 patient-years in patients treated with corticosteroids [9]. The use of csDMARDs in patients with RA may result in a higher chance of contracting a viral infection. The retrospective-population based study by Lacaille et al. demonstrates that when patients with RA are treated with csDMARD therapy, there is a small decrease in risk of mild infection (RR: 0.90, 95% CI: 0.88-0.93) and no significant association of serious infection risk (RR: 0.92, 95% CI: 0.85-1.00) [10]. So far, there has been little evidence of COVID-19 infection and its propensity to result in increased mortality or length of hospital stay in patients with RA. Due to this lack of data, the focus of our study will be to measure the degree to which COVID-19 is associated with increased mortality and length of hospital stay in patients with RA while also taking into account these patients' comorbidities.

## Materials and methods

Our retrospective study included 14,180 patients (age >18, median 58, range 18-90) who tested positive for COVID-19 or were assumed to have COVID-19 infection on admission to Hospital Corporation of America (HCA) hospitals from January 1, 2020, through July 31, 2020. Of the total 14,180 patients (males 57.1%, females 42.9%), 159 patients (1.1%), had a diagnosis of RA. Patients were grouped based on the diagnosis of RA and COVID-19 infection versus those without RA. Patients who were diagnosed with systemic lupus erythematosus (SLE), chronic obstructive pulmonary disease, and hypertension were excluded. Pregnant patients and those with no C-reactive protein (CRP) or D-dimer laboratory results were also excluded from the population. Covariates included age, body mass index (BMI), race, sex, maximum C-reactive protein value, maximum D-dimer value, and comorbid diabetes mellitus (DM). Patients recorded the use of home medications which included corticosteroids, csDMARDs, and biologic disease-modifying antirheumatic drugs (bDMARD). Outcome measures were length of hospital stay (LOS), in-hospital mortality, intensive care unit (ICU) admission, ICU LOS, mechanical ventilation, time on mechanical ventilation, and discharge to hospice. Patients' demographic and clinical characteristics are presented in Table 1. 

**Table 1:** Patients' demographic and clinical factors RA - rheumatoid arthritis; ICU - intensive care unit; DM - diabetes mellitus

Patients' demographic and clinical factors	Total number (%)
Females	6,078 (42.1%)
Males	8,102 (57.1%)
African Americans	2,963 (20.9)
Caucasians	7,205 (50.8%)
Other	4,012 (28.3%)
Without RA	14,021 (98.9%)
With RA	159 (1.1%)
Not admitted to ICU	9,631 (67.9%)
Admitted to ICU	4,549 (32.1%)
No placement on mechanical ventilation	12,167 (85.8%)
Placement on mechanical ventilation	2,013 (14.2%)
Without DM	8,421 (59.4%)
With DM	5,759 (40.6%)
Without home reported use of anti-inflammatories	13,654 (96.3)
With home reported use of anti-inflammatories	526 (3.7%)
Not discharged to hospice	13,369 (94.3%)
Discharged to hospice	811 (5.7%)
Not expired in hospital	12,400 (87.4%)
Expired in hospital	1,780 (12.6%)

Linear regression was used to determine the difference in hospital, ICU, and mechanical ventilation LOS between patients with COVID-19 infection and COVID-19 infection with RA while controlling for age, sex, race, BMI, maximum D-dimer lab value, maximum CRP lab value, and reported use of home medications. The logistic regression model was used to estimate the probability of in-hospital mortality, ICU admission, placement on mechanical ventilation, discharge to hospice, and in-hospital mortality related to home anti-inflammatory use when comparing patients with RA and COVID-19 infection to COVID-19 infected patients without RA. Continuous variables were reported as means and standard deviation (SD) or as median and range values. Categorical variables were expressed as counts and percentages. We used a chi-squared test to compare variables between COVID-19 positive patients with or without rheumatoid arthritis for categorical data. In-hospital mortality, hospital length of stay, ICU admission, ICU length of stay, mechanical ventilator usage, time on a ventilator, and discharge to hospice were calculated and adjusted for patient demographics and comorbidities through multivariate regression analysis. All statistical analyses were performed using SAS software (version 9.4; SAS Institute Inc., Cary, USA), with p<0.05 considered as statistically significant.

## Results

In-hospital mortality and LOS

The average LOS for patients with COVID-19 without RA was 8.54 days, while patients with RA and COVID-19 had an average LOS of 9.78. In the multivariate model, patients with RA diagnosed with COVID-19 have slightly longer hospital stay than those who did not have COVID-19. However, the results were not significant (β=0.011; 95% CI: 0.40-2.18; p=0.18). The proportion of COVID-19 infected patients without RA who died during their hospital stay was (12%) compared to (19%) of COVID-19 patients with RA. Patients with RA who were infected with COVID-19 were more likely to die during their hospital stay (OR=1.65; 95% CI: 1.07-2.53; p=0.02).

ICU admission and LOS

Of the total 14,180 patients in our study with COVID-19 infection, 4,549 were admitted to the ICU (32%), compared to (34%) of the total 159 patients with RA and COVID-19. The data indicates that there is no statistically significant relationship between patients with RA and COVID-19 infection and the likelihood of being admitted to ICU (β=1.03; 95% CI: 0.72-1.46; p=0.88). Patients with RA and COVID-19 spent more days on average in the ICU (9.78) compared to patients without RA (8.54). Although patients with RA, on average, stayed more days in ICU, the difference is not significant (β=0.23; 95% CI: -10.76-102.09;p=0.11).

Mechanical ventilation placement and LOS

The results illustrate that 14.1% of hospitalized patients with COVID-19 and no RA were placed on mechanical ventilation, while 22.6% of those with COVID-19 and RA required ventilation. There is statistically significant evidence that patients with RA are more likely to be placed on a ventilator than patients without RA (OR: 1.82; 95% CI: 1.22-2.71; p=0.003) or patients with RA are 1.22-2.71 times more likely to be placed on mechanical ventilation than those without RA. Patients with RA and COVID-19 are expected to spend 39.1 more hours on the ventilator than those without RA. Even though patients with RA are expected to spend more time on ventilation, the result was insignificant (β=0.02; 95% CI: -39.84-118.03; p=0.33)

Discharge to hospice and in-hospital mortality with reported use of home anti-inflammatories 

All 14,180 patients with COVID-19 infection were hospitalized, of which 180 were discharged to hospice (5.7%), as opposed to nine of the 159 total RA and COVID-19 patients (5.7%). There is no statistically significant likelihood of discharge to hospice for patients with RA and COVID-19 compared to patients without RA (OR: 0.77; 95% CI: 0.38-1.55; p=0.46). We found sufficient evidence to state that COVID-19 infected patients who report using corticosteroids and DMARDs are less likely to die in the hospital compared to those who do not report medication usage (OR: 0.64; 95% CI 0.47-0.88; p=0.007 ).

Results of the statistical analysis are presented in Table 2.

**Table 2:** Tests for differences in patients with RA and COVID-19 versus COVID-19 patients without RA RA - rheumatoid arthritis; ICU - intensive care unit

Variable	OR	β	95% confidence interval	p-value
Length of hospital stay	-	0.11	-0.40-2.18	0.18
In-hospital mortality	1.65		1.08-2.54	0.02
ICU admission	1.03		0.72-1.46	0.88
Length of stay in ICU	-	0.11	-10.76-102.09	0.11
Mechanical ventilation	1.82		1.22-2.71	0.003
Length of stay on mechanical ventilation	-	0.33	-39.84-118.03	0.33
Discharged to hospice	0.77		0.38-1.55	0.46

## Discussion

The objective of our retrospective study was to determine if patients diagnosed with COVID-19 and RA would be predisposed to poor outcomes, including increased hospital LOS, mortality, ventilator use, time on a ventilator, ICU admission, time in ICU, or discharge to hospice compared to patients with only COVID-19 infection while controlling for age, sex, race, BMI, DM, maximum D-dimer lab value, maximum CRP lab value, and reported use of home medications. After analyzing the data of 14,180 patients with COVID-19 infection, it can be determined that patients with RA will have an increased risk for poor outcomes in hospital in terms of mortality, ICU length of stay, and likelihood to be placed on mechanical ventilation. Our study indicates that patients diagnosed with RA and COVID-19 are 1.65 times more likely to die in hospital than those without RA. The same patients are 1.82 times more likely to be placed on mechanical ventilation than those without an RA diagnosis. Although not statistically significant, we found that RA might hold clinical significance as those patients were noted to spend longer in the ICU as well as on mechanical ventilation compared to those without RA, 39.09 hours and 45.76 days, respectively.

It is important to note that patients with reported use of home medications, such as DMARDs and corticosteroids, were less likely to experience hospital mortality. Our study demonstrated that patients with reported use of anti-inflammatory medications had a statistically significantly decreased risk of in-hospital mortality compared to patients who did not report using those drugs. The decreased risk of mortality in this group of patients could be due to superior control of the destructive inflammatory response often seen in patients with severe COVID-19 infection. An example of anti-inflammatory control of the COVID-19 inflammatory response is illustrated in the RECOVERY group study, which tests the use of dexamethasone in COVID-19 infected patients requiring oxygen or mechanical ventilation. The incidence of death in COVID-19 infected patients on mechanical ventilation was much lower in patients receiving dexamethasone compared to the usual care (29.3% vs. 41.4%; RR: 0.64; 95% CI: 0.51-0.81), as well as infected patients requiring oxygen 23.3% vs. 26.2%; RR: 0.82; 95% CI: 0.72-0.94) [11]. However, when answering the question of whether RA patients being preemptively treated with DMARDs will result in less incidence of COVID-19 infection, we see in the study by Favalli et al. that the incidence is consistent with the general population (0.62% vs. 0.66%; p=0.92) [12].

In the peer-reviewed literature review, few studies have explored the relationship between patients with RA and COVID-19 infection and their propensity to result in suboptimal hospital outcomes. One study in particular that came close to describing this relationship was the OpenSAFELY study by Williamson and colleagues [5]. The study utilized the OpenSAFELY electronic medical record (EMR), which contained the electronic record of over 17 million patients in England. They analyzed the data to determine factors related to COVID-19 mortality. The authors of the study analyzed patients with the inflammatory conditions RA, systemic lupus erythematosus (SLE), and psoriasis as one group. They found that while controlling for age, sex, and chronic health conditions, patients with these illnesses and COVID-19 infection had a slightly increased risk of hospital mortality (adjusted hazard ratio [HR]: 1.19; 95% CI: 1.11-1.27). While the study does not answer the question of whether patients diagnosed with RA and COVID-19 specifically experience increased hospital mortality, it does provide a point of reference for inflammatory conditions [5].

Comparing the study results by Williams and colleagues to ours, we see similar results with in-hospital mortality. While controlling for confounding variables, both of the studies demonstrate a statistically significant increased likelihood of patients with inflammatory conditions to expire in a hospital with COVID-19 compared to patients with only COVID-19 infection. It must be noted that the Williams study is analyzing COVID-19 patients with not only RA but also psoriasis and SLE before comparing them to patients with only COVID-19 infection. The 17,278,392 pool of patients in the Williams and colleagues study is also substantially larger compared to our pool of 14,180 patients.

Some of our study limitations include a relatively small sample size of our population of patients with RA and COVID-19, resulting in 159 patients. Secondly, another potential drawback was that although rheumatoid factor (RF) and anti-cyclic citrullinated peptide (anti-CCP) were obtained, these values were not systematically checked for every patient admitted with RA. It was difficult to distinguish whether these patients were seronegative or seropositive as no outpatient data was obtained. Finally, given that our data was acquired during a relatively early period of the pandemic (01/01/20 - 07/31/20) when vaccines were not available, we did not take into account the protective effect of COVID-19 vaccination on patients with RA. 

Despite our study limitations, a potential implication would be to include RA as established comorbidity and an independent risk factor that could result in a higher likelihood of COVID-19 related patient morbidity or mortality. Therefore, it could be even more vital for this patient population to receive vaccination, as the phase III placebo-controlled trial for Pfizer vaccine by Polak et al. illustrates that the vaccine provides 95% efficacy when preventing COVID-19 symptoms at least seven days after the second dose [13].

## Conclusions

This study suggests that patients with RA and COVID-19 have a significantly increased likelihood of in-hospital mortality and placement on mechanical ventilation. While challenging to determine in a pandemic situation, large nationwide studies are necessary to improve our understanding of COVID-19 infection in patients diagnosed with RA. A potential follow-up study could be to investigate how the prophylactic use of anti-inflammatory medications can possibly reduce in-hospital mortality of patients who eventually become infected with COVID-19.
